# Use of the Kwak Thyroid Image Reporting and Data System (K-TIRADS) in differential diagnosis of thyroid nodules: systematic review and meta-analysis

**DOI:** 10.1007/s00330-017-5230-0

**Published:** 2018-01-02

**Authors:** Bartosz Migda, Michal Migda, Marian S. Migda, Rafal Z. Slapa

**Affiliations:** 1 Diagnostic Imaging Department, Medical University of Warsaw, Kondratowicza 8, 03-242 Warsaw, Poland; 2 Clinical Unit of Obstetrics, Women’s Disease and Gynaecological Oncology, United District Hospital, Collegium Medicum, University of Nicolaus Copernicus, sw. Jozefa 53-59, 87-100, Torun, Poland; 3 Civis-Vita Health and Medical Centre, Warszawska 20, 87-100, Torun, Poland

**Keywords:** Thyroid nodules, Thyroid neoplasm, Ultrasonography, Risk assessment, Meta-analysis

## Abstract

**Purpose:**

The purpose of this systematic literature review was to assess the usefulness of the Thyroid Image Reporting and Data System (K-TIRADS) classification proposed by Kwak for differentiation of thyroid nodules.

**Material and methods:**

Four literature databases were searched for relevant articles through early January 2017. A meta-analysis was performed to calculate pooled sensitivity, specificity, positive likelihood ratio (LR+), negative likelihood ratio (LR-) and diagnostic odds ratio (DOR). The area under the curve (AUC) from the pooled receiver operating characteristic (ROC) was used to assess the usefulness of this classification for differentiation of thyroid nodules. Meta-analysis was conducted by using meta-analysis software.

**Results:**

We analysed six publications describing 10,926 nodules. Pooled sensitivity, specificity, LR+, LR-, DOR, and AUC for pooled ROC were 0.983 (95 % CI 0.976–0.989), 0.552 (95 % CI 0.542–0.562), 2.666 (95 % CI 1.692–4.198), 0.05 (95 % CI 0.035–0.072), 51.020 (95 % CI 15.241–170.79) and 0.938, respectively.

**Conclusions:**

Kwak TIRADS has high sensitivity and low specificity. Thus, it is very useful to discard the benign cases and to reduce the number of biopsies.

**Key Points:**

*• Routine, adequate standardization of thyroid nodules ultrasound classification is mandatory.*

*• Kwak TIRADS parameters are accurate for differentiating focal thyroid lesions.*

*• Kwak TIRADS system is simple to apply.*

*• Kwak TIRADS system may become a useful diagnostic tool.*

## Introduction

Ultrasound imaging is a basic technique used in the visualization and characterization of focal thyroid lesions and to estimate the risk of malignancy. The suspicious nature of lesions is confirmed on the basis of cytological examination of specimens collected via ultrasound-guided fine-needle aspiration biopsy (FNAB) and further histological examination if necessary [1]. Currently, ultrasound and FNAB serve as essential tools for diagnosing thyroid nodules [1, 2]. Numerous studies have shown that ultrasonography has an important place in the diagnosis of malignant and benign thyroid lesions and is marked by high sensitivity and low specificity [3]. Many lesions without suspicious features may be observed conservatively in this manner without the necessity of biopsy [2, 4].

Currently, the fundamental technique used for clinical assessment of thyroid lesions is grey-scale imaging (B-mode). In B-mode, suspicious features include: solid nature; low or very low echogenicity; irregular, microlobular or blurred borders; vertical shape or an anteroposterior diameter greater than lateral; and microcalcifications [2, 5, 6].

There are numerous guidelines of many medical societies worldwide that describe the clinical and ultrasound features that necessitate FNAB [7–9]. However, there is a need for application of routine, adequate and common standardization system of thyroid nodules ultrasound classification. The system has already been proposed and is called Thyroid Imaging Reporting and Data System (TIRADS). It relies on B-mode imaging and represents an important step in standardization of ultrasound examination of the thyroid. TIRADS has its foundation in the Breast Imaging Reporting and Data System (BIRADS) classification [10–12], which is based on varying, increasing the risk of malignancy of focal lesions in different categories. Data relating to TIRADS classification were first published in 2009 by two independent teams led by Horvath [13] and Park [14]. The two different approaches proposed by the teams, in our opinion, proved to be complicated and difficult to use on a daily basis. The later study published by Kwak in 2011 [6] had a different approach to this classification. Whether the individual lesion belongs to a TIRADS category or not was determined based on the number of suspicious features, including solid structure, low or very low echogenicity, irregular or microlobular borders, microcalcifications and vertical shape (TIRADS 3 = no suspicious features; TIRADS 4a = 1 suspicious feature; TIRADS 4b = 2 suspicious features; TIRADS 4c = 3 or 4 suspicious features; TIRADS 5= 5 suspicious features).

Furthermore, other groups proposed different TIRADS interpretations [15–17].

The aim of this study was to conduct a systematic literature review and to assess the diagnostic utility of the Kwak’s TIRADS classification in the risk stratification of thyroid nodules in adults.

## Materials and method

### Eligibility criteria

The described systematic review of the literature and meta-analysis were carried out in accordance with the recommendations of the Preferred Reporting Items for Systematic Reviews and Meta-Analysis (PRISMA) [18]. The studies were included in the analysis based on the following criteria (participants, interventions, comparators, outcomes, and study design (PICOS) questions): adults with focal thyroid changes; TIRADS classification as proposed by Kwak used in differential diagnosis of thyroid nodules; reference examinations were histological and/or cytological, ultrasound follow-up lasting more than 12 months; retrospective and prospective studies published in English or German. The full text of duplicated publications was used to obtain more precise data necessary for analysis.

### Data sources

We searched four databases (PubMed, Cochrane database, ScienceDirect and EMBASE) from January 2009 to January 2017. The following terminology was adopted: ‘TIRADS’ OR ‘TI-RADS’ OR ‘thyroid imaging reporting and data system’ OR ‘reporting system for thyroid nodules’. Subsequently, the titles of studies and abstracts were validated for inclusion of the object of the analysis. Full versions of relevant articles were then downloaded for further analysis. Full text articles were examined for inclusion criteria by two independent reviewers (B.M. and M.S.M.). The reference list of obtained publications was then manually checked to identify other studies related to the topic.

### Inclusion criteria

For inclusion in the present study, patients had to meet all of the following criteria: (1) adults with thyroid nodules, including patients with nodular goitre; (2) differential diagnosis used the TIRADS classification proposed by Kwak; (3) data contained in publications had to allow performing calculations in 2 x 2 pivot tables; (4) conclusive diagnosis was established on the basis of histopathological and/or cytological examination or ultrasound follow-up longer than 12 months in case of benign nodules; (5) the patient must not have been a group subject in previous studies. If samples were part of previously published material, the data from such studies were used to obtain more accurate information on the study group. The decision to classify the study was made independently by two authors (B.M. and M.M.). Discrepancies were resolved by consensus, which occurred in four cases of 42 full-text articles.

### Data extraction

The extracted data included authors, country of origin, patient group data (size of group, sex distribution and average age/range of age), number of nodules, study design (prospective, retrospective), reference method and study results. To qualitatively assess the methodology of analysed publications, the widely used and recognized Quality Assessment of Diagnostic Accuracy Studies (QUADAS-2) tool was used [19].

### Statistical analysis and data synthesis

A random effects model that assumes statistical heterogeneity of study results was used in the meta-analysis. Sensitivity, specificity, positive predictive value (PPV), negative predictive value (NPV), diagnostic accuracy (ACC) and odds ratio (OR) for each study was calculated using 2 x 2 pivot tables. The Spearman correlations coefficient was used to carry out the threshold analysis for the index test. Heterogeneity was assessed using probabilities of the Chi^2^ (χ^2^) test by reporting the I^2^ statistics, which is independent of the number of studies in the meta-analysis. The I^2^ value varies from 0 % to 100 %, where 0 % means no heterogeneity between studies and values greater than 50 % indicate a significant heterogeneity.

After the assessment of heterogeneity, the following pooled values were calculated: sensitivity, specificity, positive likelihood ratio (LR+), negative likelihood ratio (LR-) and diagnostic odds ratio (DOR). A forest plot was generated for each value. In addition, a funnel plot was used to assess the possibility of errors in publication and statistics using the Egger, Begg and Mazumdar tests to assess the significance of funnel plot asymmetry. The diagnostic usefulness of Kwak’s TIRADS classification was assessed using the summarized receiver operating characteristic (ROC) curve computed via the DerSimonian-Laird random effects model. The area under the curve (AUC), standard error (SE) for AUC, Q* statistics and its SE are reported.

In the case of high heterogeneity, meta-analyses were performed in subgroups and via univariate meta-regression. Grouping variables included the year of publication (before or after 2016), the number of nodules (more or less than 1,000), the country (Korea vs. other), the reference type (cytology and histology vs. cytology, histology and ultrasound follow-up), the cut-off (K-TIRADS category 3/4a vs 4a/4b) and the type of study (retrospective vs. prospective).

P-values less than 0.05 were considered significant. Statistical analyses were conducted using Statistica 13.1 (StatSoft Inc.) and MetaDisc (Freeware Software).

## Results

### Selection and data extraction

**Fig. 1:**
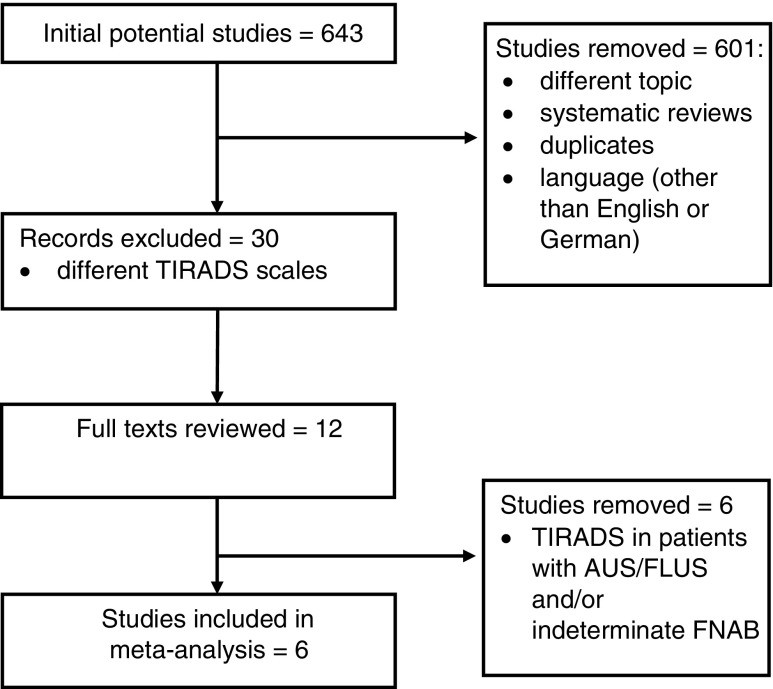
Literature search and selection. *TIRADS* Thyroid Image Reporting and Data System, *AUS/FLUS* atypia of undetermined significance/follicular lesion of undetermined significance, *FNAB* fine needle aspiration biopsy

**Fig. 2:**
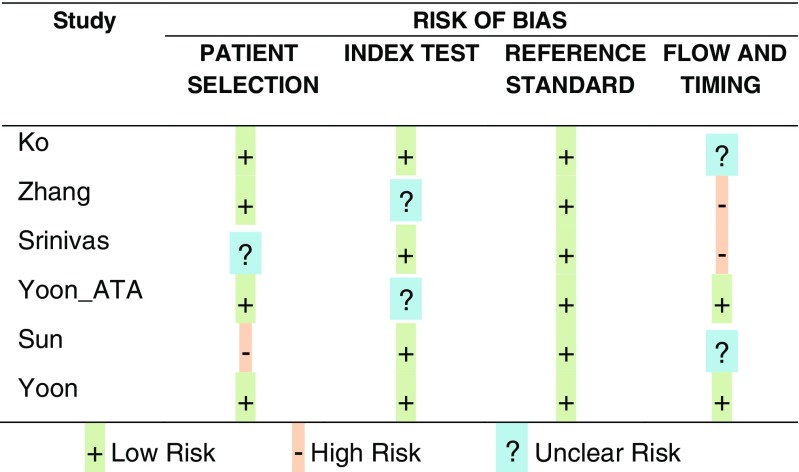
Results of QUADAS-2 assessment for risk of bias in individual studies

A total of 643 publications were identified upon systematic review of the literature, of which 42 remained after removing duplicates, literature reviews, studies not related to Kwak’s TIRADS and those written in a language other than English or German. Analysis of abstracts and full texts allowed isolation of six original studies that assessed the K-TIRADS scale proposed by Kwak, consistent with the meta-analysis objectives [20–25] (Fig. 1). The six studies that referred to K-TIRADS in patients with AUS/FLUS or indeterminate cytology were excluded [26–31]. A total of 10,926 nodules were included in the meta-analysis. QUADAS-2 classification (Fig. 2) [19], funnel graphs and tests verifying asymmetry were used to assess the quality of included publications.

**Fig. 3:**
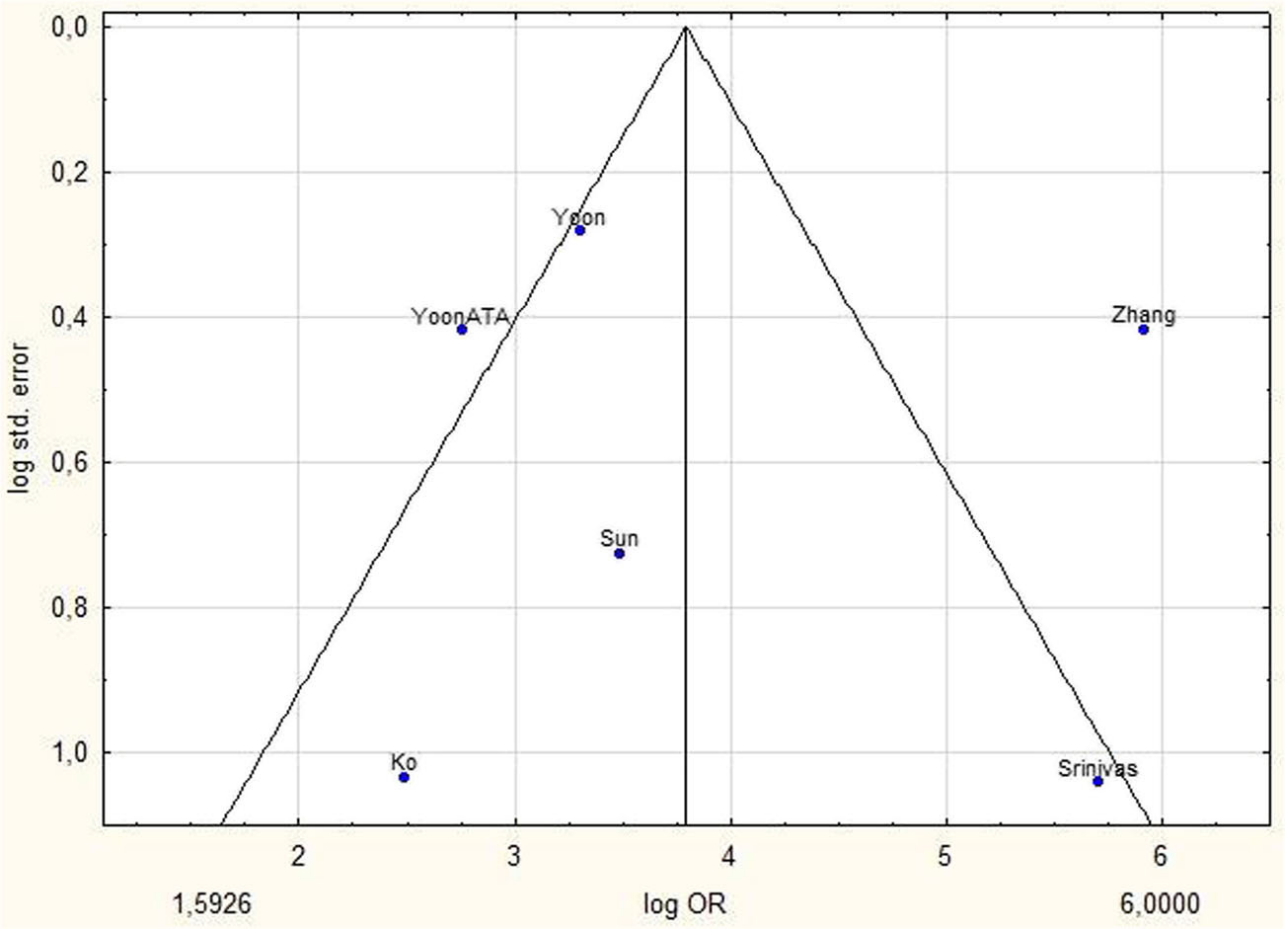
Funnel plot shows log standard error by log OR for Kwak’s TIRADS. P*-*value calculated with Begg and Mazumdar test 0.6015 and Egger test 0.7783 indicates that there was no substantial publication bias. *TIRADS* Thyroid Image Reporting and Data System

Analysed studies were published between 2014 and 2017. There were two prospective and four retrospective studies. The number of nodules in each publication varied between 204 and 4,696. The average patient age was 47.8 years. In four of six studies, cytological and histological examinations were used for verification. In the study by Zhang and Sun, ultrasound check was also taken into account for TIRADS 2 category lesions with initial benign cytology [21, 25] (Table 1). The cut-off value in four studies was category 4a/4b [20, 22–24] and in last two studies category 3/4a [21, 25]. The funnel graph for analysed studies was asymmetrical, which may suggest publication bias (Fig. 3). Begg, Mazumdar and Eggar tests were used to assess the significance of asymmetry. Finally, the magnitude of the calculated effect in each of the six studies was not strongly enough associated with standard error for tests to confirm the observed asymmetry. None of the tests showed a relationship between the values ​​of effect and their precision (P = 0.6015 by Begg and Mazumdar test, P = 0.7783 by Egger test).

**Table 1:** Baseline characteristics of included studies

Study	Year of publication	Country	Patients	Nodules	Average age	Study design	Reference standard	Sensitivity	Specificity	PPV	NPV	ACC
Ko et al. [20]	2014	Korea	195	204	51	retrospective	Cytology and histology	0.985	0.158	0.354	0.957	0.422
Zhang et al. [21]	2015	China	2,921	3,980	51.6	prospective	Cytology, histology and US follow-up	0.974	0.91	0.396	0.998	0.913
Srinivas et al. [22]	2016	India	365	365	33.1	prospective	Cytology and histology	0.96	0.926	0.49	0.997	0.929
Yoon ATA et al. [24]	2016	Korea	1,241	1,293	50.8	retrospective	Cytology and histology	0.974	0.293	0.233	0.981	0.416
Sun et al. [25]	2017	China	1,293	388	49.5	retrospective	Cytology, histology, and US follow-up	0.987	0.307	0.473	0.973	0.57
Yoon et al. [23]	2016	Korea	4,585	4,696	50.9	retrospective	Cytology and histology	0.988	0.255	0.275	0.986	0.418

*PPV* positive predictive value, *NPV* negative predictive value, *ACC* diagnostic accuracy

**Fig. 4:**
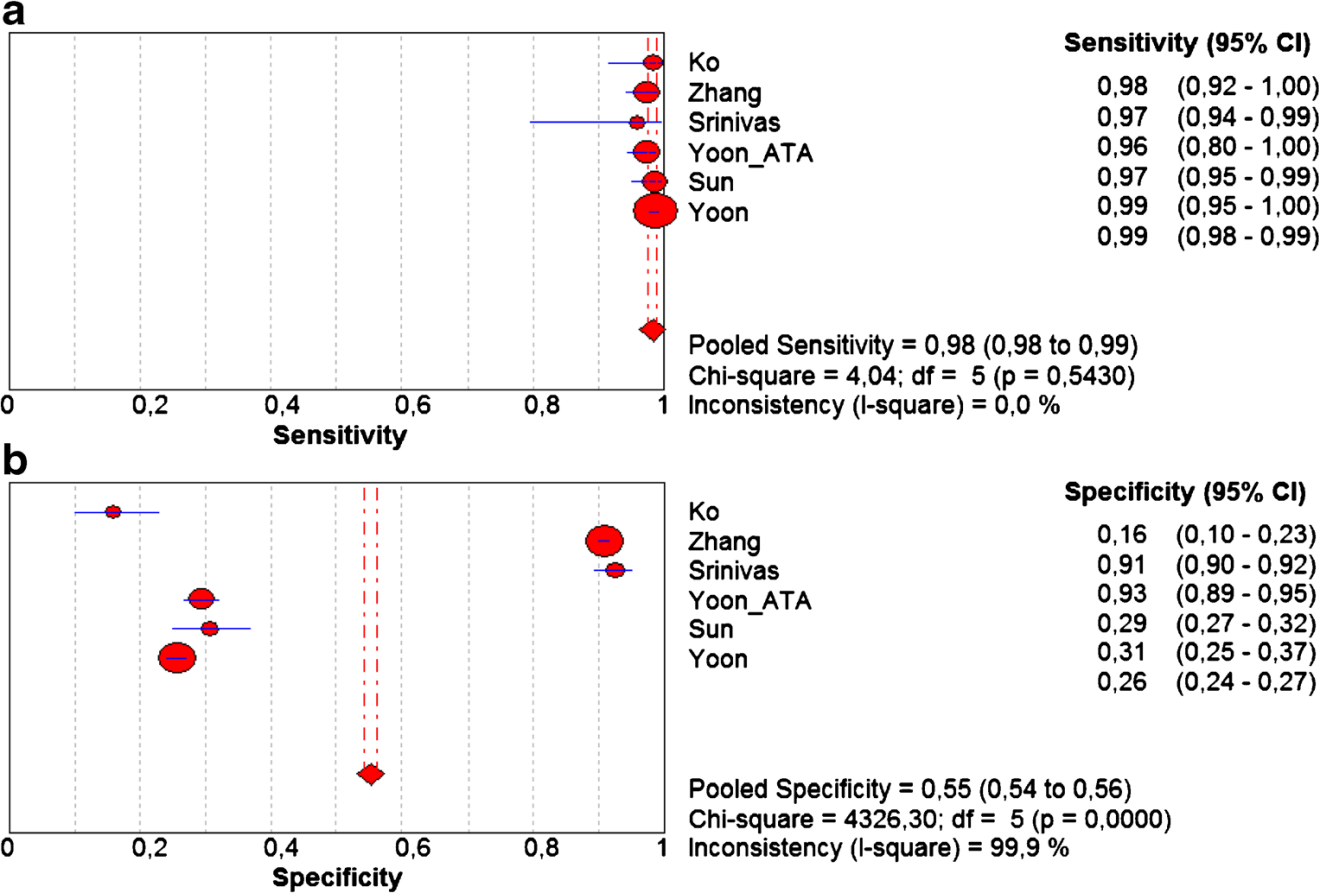
Forest plots of eligible studies showing individual and pooled (**A**) sensitivities and (**B**) specificities of Kwak’s TIRADS in the differential diagnosis of thyroid nodules. Pooled values indicated by diamonds with 95 % confidence intervals (in brackets). *TIRADS* Thyroid Image Reporting and Data System

**Fig. 5:**
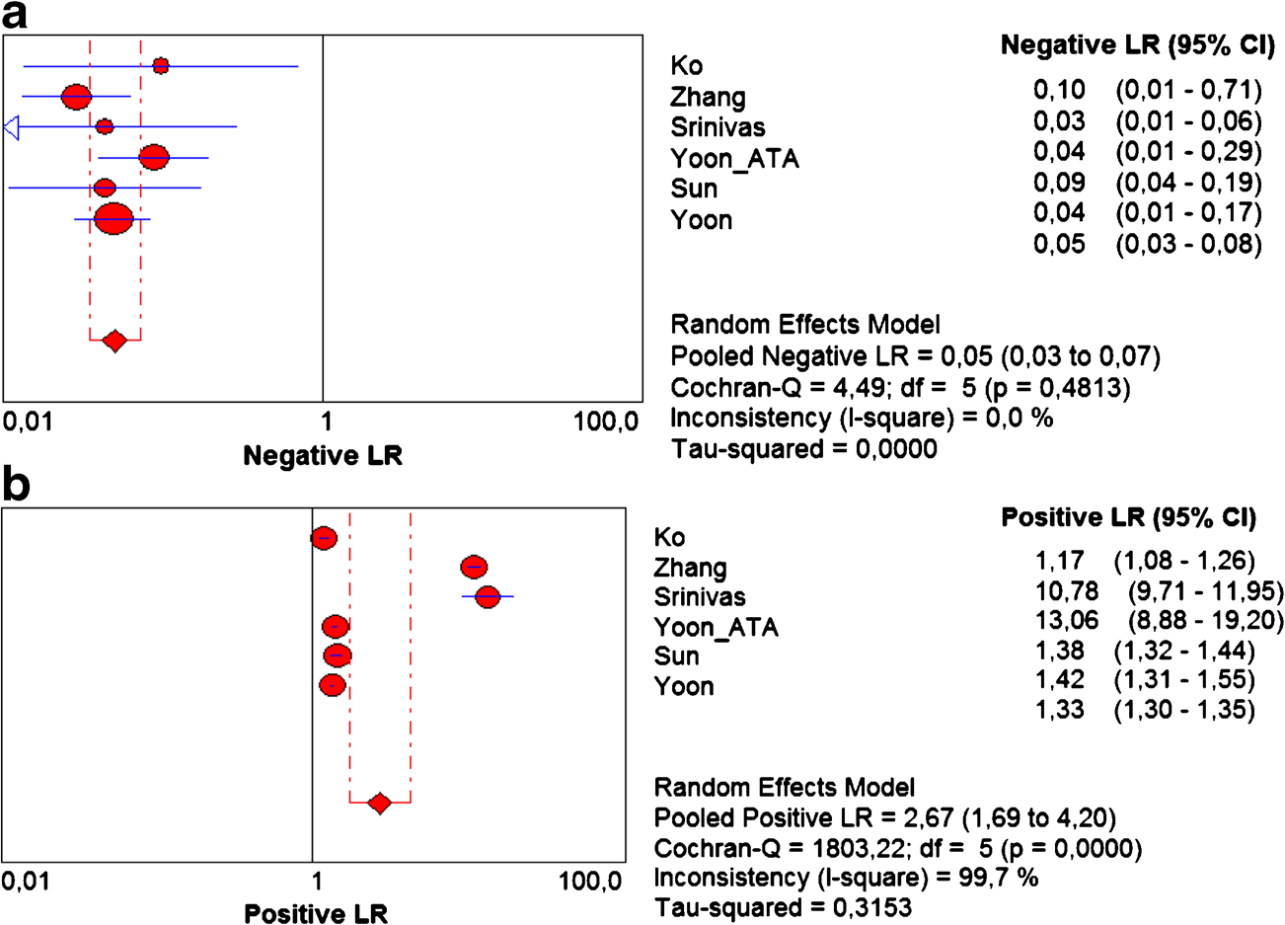
Forest plots of eligible studies showing individual and pooled (**A**) negative and (**B**) positive likelihood ratios (LRs) of Kwak’s TIRADS in the differential diagnosis of thyroid nodules. Pooled values indicated by diamonds with 95 % confidence intervals (in brackets). *TIRADS* Thyroid Image Reporting and Data System

**Fig. 6:**
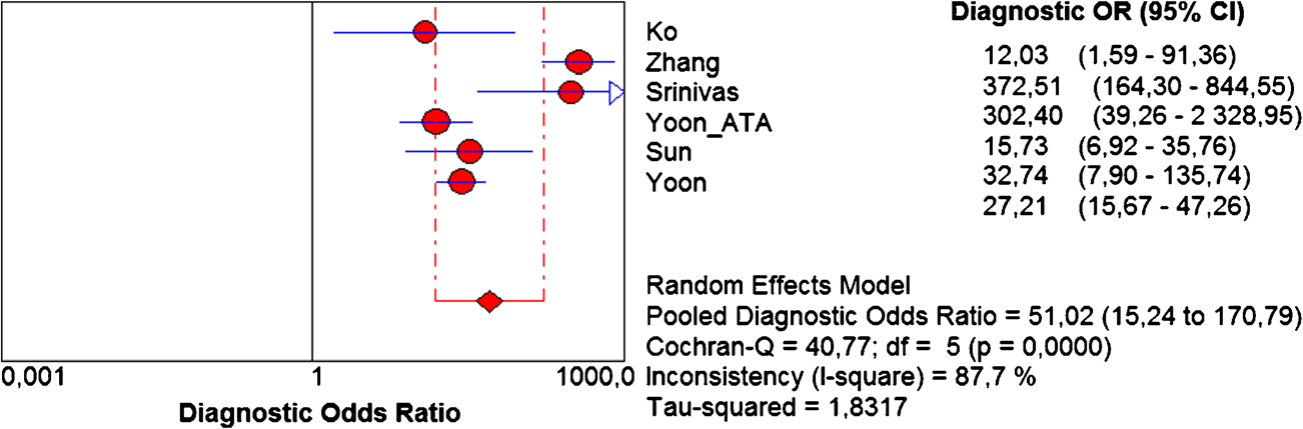
Forest plot of eligible studies showing individual and pooled diagnostic odds ratio (DOR) of Kwak’s TIRADS in the differential diagnosis of thyroid nodules. Pooled value marked by diamonds with 95% confidence interval (in brackets). *TIRADS* Thyroid Image Reporting and Data System

The pooled sensitivity and specificity for K-TIRADS classification were 0.983 (95 % CI 0.976–0.989) and 0.552 (95 % CI 0.542–0.562), respectively (Fig. 4 a,b). Sensitivity was very high in all studies ranging from 0.972 to 0.985. Specificity was more varied and ranged from 0.261 to 0.911. Pooled values of positive likelihood ratio (LR+) and negative likelihood ratio (–LR-) were 2.666 (95 % CI 1.692–4.198) and 0.05 (95 % CI 0.035–0.072), respectively (Fig. 5 a,b). Pooled diagnostic odds ratio (DOR) was 51.020 (95 % CI 15.241–170.79) (Fig. 6). The area under the ROC curve was equal to 0.938 and the Q* statistic was 0.8772, confirming a very good diagnostic accuracy of the analysed test.

### Analysis in subgroups and meta-regression

Meta-regression was performed in four subgroups, of which heterogeneity was found in two (country of origin p = 0.0037 and study design p < 0.0001). Both subgroups had a significant impact on the heterogeneity of this meta-analysis. The relative DOR for the country of origin was 2.14, while for study design it was 78.61 (Table 2). These results demonstrate that the diagnostic accuracy of K-TIRADS classification in prospective studies was higher than in retrospective studies. Moreover, the studies from outside of Korea had a greater effect on pooled sensitivity and specificity (p < 0.05).

**Table 2:** Subgroup analysis

Subgroup		No. of studies	Pooled sensitivity (95 % CI)	Pooled specificity (95 % CI)	Pooled DOR	Meta-regression RDOR	Meta-regression (p-value)
Total		6	0.983 (0.976–0.989)	0.552 (0.542–0.562)	51.020		
Publication year	< 2016	2	0.976 (0.951–0.990)	0.883 (0.872–0.893)	75.907		
	≥ 2016	4	0.985 (0.977–0.990)	0.308 (0.296–0.321)	31.260	0.71	0.2701
No. of nodules	< 1000	4	0.983 (0.958–0.995)	0.572 (0.535–0.608)	47.350		
	≥ 1000	2	0.983 (0.976–0.989)	0.550 (0.539–0.561)	53.742	0.98	0.9301
Korea vs. other	Korea	3	0.985 (0.977–0.991)	0.261 (0.249–0.273)	22.237		
	Other	3	0.978 (0.958–0.990)	0.878 (0.868–0.887)	156.13	2.14	0.0037
Reference standard	Cytology, histology	4	0.971 (0.961–0.980)	0.392 (0.379–0.405)	49.665		
	Cytology, histology and US follow-up	2	0.979 (0.959–0.991)	0.874 (0.863–0.884)	118.31	0.36	0.4241
Cut–off	3/4a	2	0.979 (0.959–0.991)	0.874 (0.863–0.884)	118.31		
	4/4b	4	0.971 (0.9610.980)	0.392 (0.379–0.405)	49.665	0.36	0.4241
Study design	Prospective	2	0.972 (0.944–0.989)	0.911 (0.902–0.920)	361.91		
	Retrospective	4	0.985 (0.978–0.991)	0.263 (0.251–0.275)	23.023	78.61	<0.0001

*CI* confidence interval, *DOR* diagnostic odds ratio, *RDOR* relative diagnostic odds ratio

Analysed differences in cut-off point and reference standard did not revealed significant importance (p>0.05).

## Discussion

Ultrasonography is considered to be the test of choice in preoperative diagnosis of thyroid nodules [6]. There is no doubt that the coexistence of a greater number of suspicious features in a focal lesion significantly increases the risk of malignancy compared to a single suspicious feature. At the same time, the lack of a unified system for categorizing nodules often caused misunderstandings between radiologists and clinicians. The proposed TIRADS classification was intended to be a response to these needs. A large number of studies have been published evaluating the discriminatory capabilities of TIRADS based on different classifications [13, 32–34] and recent WFUMB (World Federation for Ultrasound in Medicine and Biology) guidelines have suggested using TIRADS in order to improve characterization of thyroid nodules, and especially the communication between specialists and patients [35].

Previous meta-analyses evaluating the diagnostic utility of the TIRADS classification took into account its different variants, which could significantly affect the results obtained. In Wei's 2016 study, reported pooled sensitivity and specificity were 0.79 and 0.71, respectively, and for AUC was 0.92 for the summarized ROC. The results of the current meta-analysis including six studies and a total of 10,926 nodules show that the classification proposed by Kwak has a much higher pooled sensitivity of 0.983 and lower specificity of 0.552 in differentiation of thyroid nodules, compared to the previous meta-analyses [36, 37]. Other parameters, such as pooled positive likelihood ratio (2.67, 95 % Cl 1.69–4.2) and negative likelihood ratio (0.05, 95 % Cl 0.03–0.07), were slightly lower than those reported in Wei's study, while the diagnostic odds ratio of 51.02 (95 % CI 15.24–170.79) was considerably higher. Apart from these differences, the parameters also indicate good diagnostic value of the TIRADS classification proposed by Kwak.

The initial assessment of obtained studies suggested heterogeneity, which after more detailed analysis turned out to be statistically insignificant with p > 0.05. Exploration of possible causes revealed that important factors that may increase the heterogeneity of results include study type and country of origin. Analysis of subgroups showed a statistically significant difference between retrospective and prospective studies with p < 0.0001 and significantly higher values of diagnostic odds ratio in prospective studies (361.91 vs. 23.023 for retrospective studies). Further analysis demonstrated that in prospective studies, the pooled sensitivity was lower than in the retrospective study group (0.972 vs. 0.985). The pooled specificity was significantly higher (0.911 vs. 0.263). The results of prospective studies that verify nodules by means of cytological and histopathological evaluation better reflect everyday practice than retrospective studies, which classify material via histopathological verification.

Another essential feature impacting the heterogeneity of results was the country of origin. It is worth noting that three of the six studies originated from Korea, one was from India, and two were from China. Subgroup analysis demonstrated a statistically significant difference between the studies originating from Korea versus other countries (p = 0.0244). In China, cytological verification of focal lesions is rarely performed and thyroid cancer is confirmed mainly on the basis of histological examination [37]. The result is that in the case of patients with nodules classified as TIRADS 1–3 and first cytological, non-diagnostic, atypical or follicular lesion of undetermined significance (Bethesda categories I and III), the invasive diagnostics were not enhanced with another aspiration biopsy or surgery. As a consequence, this causes a lack of diagnosis of cancer in these TIRADS categories and negatively affects the assessment of specificity for this classification [37].

In analysed publications the cut-off point was different. In two cases the cut-off was 3/4a, while in the remaining four it was 4a/4b (Table 2). There is a high difference in specificity, which is higher for 3/4a 0.874 (95 % CI 0.863–0.884) compared to 4a/4b 0.392 (95 % CI 0.379–0.405), when sensitivity for 3/4a 0.979 (95 % CI 0.959–0.991) is almost parallel to 4a/4b 0.971 (95 % CI 0.961–0.980). Similar results were obtained for accepted reference methods. In two cases authors used cytology, histology and ultrasound follow-up for non-suspicious lesions in ultrasound examination or after initial benign cytology (Table 2). In both cases accepted cut-off differences and reference standards did not significantly influence homogeneity of pooled data p > 0.05 (Table 2).

In our results comparing French-TIRADS we report slightly lower specificity, 0.552 versus 0.61, but sensitivity was higher, 0.983 versus 0.957 [15]. The specificity difference could be the consequence of implementation of elastography in French-TIRADS. Obtained pooled data turned out to be lower compared to Horvath’s recent work from 2016 reporting sensitivity of 0.996 and specificity of 0.744. A recently published work on TI-RADS by ACR (American College of Radiology) proposed a different approach [38]. The study is based on a previously published ACR Thyroid Ultrasound Reporting Lexicon [39]. The authors proposed a five-grade scale of TR1 (benign) to TR5 (high suspicion of malignancy) based on scoring of five nodule characteristics (composition, echogenicity, shape, margin and echogenic foci). It was noted that due to the lack of elastography in each ultrasound scanner, it was not included in the ACR TI-RADS. This system needs to be verified as a tool in thyroid nodule stratification.

To minimise the erroneous selection of publications in the course of this systematic review, four databases were used: PubMed, Cochrane database, ScienceDirect and EMBASE. In addition, in order to assess the quality of publications the updated version of the QUADAS-2 tool was used [19].

This systematic review also has some limitations. First, in some studies it was not clearly established if the interpretation of ultrasound images of focal lesions on the basis of tested K-TIRADS classification was carried out without knowledge of the results of reference tests and vice versa. At this level, we cannot clearly determine whether this error resulted from incorrect planning of the original study methodology or from inadequate reporting. Currently, it is recommended that people studying diagnostic accuracy use the Standard for Reporting Diagnostic Accuracy check-list to minimize errors in publication of results [40]. Second, the final diagnosis was not always established on the basis of histopathological examination. Patients in whom lesions were classified as categories 1–3 were included into routine ultrasound follow-up similarly, and not in every case of a focal lesion with category 4–5 was histopathological verification performed; some diagnoses were established on the basis of cytological examination. Third, some articles found in the database searches were rejected as they were in a language other than those approved in the study protocol. Fourth, the disadvantage of previous studies is the small number of non-papillary carcinomas, which often present with a different appearance on the ultrasound examination. Particularly interesting is the group of follicular lesions of indeterminate cytology in which elastography may be useful in differentiating benign and malignant nodules [41]. The creation of a final, comprehensive, ultimate TIRADS classification in the future should include evaluation of a significant number of less prevalent non-papillary cancers as their proper diagnosis is a very important issue from a medical point of view.

## Conclusions

K-TIRADS classification has high sensitivity and low specificity. Thus, it is very useful to discard the benign cases and to reduce the number of biopsies. Further prospective, multicentre studies are needed in order to systematize this classification.

## Abbreviations and acronyms

ACC: Accuracy
ACR: American college of radiology
AUS/FLUS: Atypia of undetermined significance/follicular lesion of undetermined significance
B-mode: Grey-scale imaging
DOR: Diagnostic odds ratio
FNAB: Fine-needle aspiration biopsy
LR-, LR+: Negative, positive likelihood ratio
PICO: Patient, problem, population; intervention, cause, or prognosis; comparison or control; outcome
PRISMA: Preferred reporting items for systematic reviews and meta-analysis
QUADAS: Quality assessment of diagnostic accuracy studies
SE: Standard error
WFUMB: World federation for ultrasound in medicine and biology
